# KIAA1549-BRAF Gene Fusion Spindle Cell Sarcoma With Infantile Fibrosarcoma-Like Pattern in a Pediatric Patient: A Case Report

**DOI:** 10.7759/cureus.51981

**Published:** 2024-01-09

**Authors:** Rawan A Abualola, Tariq Al-Zaid

**Affiliations:** 1 Anatomic Pathology, King Fahad Medical City, Riyadh, SAU; 2 Anatomic Pathology, King Faisal Specialist Hospital and Research Centre, Riyadh, SAU

**Keywords:** cytogenetics, mesenchymal, pediatric, fibrosarcoma, infantile, kiaa1549-braf, sarcoma, spindle cell

## Abstract

Spindle cell sarcoma with *KIAA1549-BRAF* fusion is a type of childhood sarcoma that closely resembles infantile fibrosarcoma by morphologic criteria and harbors molecular alteration other than the *ETV6-NTRK3* fusion gene. This neoplasm was diagnostically challenging without molecular tests, including next-generation sequencing. The discovery of *BRAF* translocation in this tumor contributes to the promise of the clinical implication of selecting new therapeutic options for the treatment of progressive diseases that are refractory to conventional chemotherapy. Here we present the case of a three-year-old girl who was diagnosed with spindle cell sarcoma with *KIAA1549-BRAF* fusion gene and had a favorable outcome three years after surgery.

## Introduction

Childhood soft-tissue sarcomas are a broad group of cancers that can develop in various anatomic locations, and their cell of origin is typically mesenchymal. Soft tissue tumors are generally uncommon and affect about 7% of the pediatric group [1].

Some pediatric sarcomas exhibit a spindle cell morphology in a fascicular growth pattern, which is a feature of infantile fibrosarcoma (IFS). IFS is a rare primitive fibroblastic neoplasm that most frequently arises in the soft tissues during infancy and early childhood [2]. IFS has a favorable outcome with conservative surgical resection being the preferred therapy and/or cytotoxic chemotherapy [3].

In the last two decades, cytogenetic and molecular analyses have established that the majority of infantile fibrosarcomas harbor a characteristic translocation between chromosomes 12 and 15 which results in the *ETV6-NTRK3* gene fusion [4]. Recently, a subset of IFS lacks the* ETV6-NTRK3* fusion and a new spindle cell tumor has been described based on other molecular abnormalities. Next-generation sequencing (NGS) has found a significant number of fusion genes in soft tissue sarcomas [5]. It is now demonstrated that tumors with IFS-like morphology could harbor gene fusions involving additional related kinase genes, such as *NTRK1/2, MET,* and *RET* [6]. Novel therapies can target several of these molecular changes, with clinical implications in patients with IFS-like spindle cell sarcomas that have these mutations [7].

BRAF gene fusions are one of these new discoveries with novel fusion partner *KIAA1549*. In 2018, Kao et al. reported five cases of pediatric spindle cell sarcomas with *BRAF* gene rearrangement [5]. Penning et al. reported the first case of pediatric spindle cell sarcoma with *KIAA1549-BRAF* gene fusion in 2021 [8]. Recently, two cases of pediatric spindle cell sarcoma with *KIAA1549-BRAF* gene rearrangement resembling IFS morphologically were described [9,10]. To our knowledge, this is the fourth case to be reported of spindle cell sarcoma with KIAA1549-BRAF gene fusion in a female child.

## Case presentation

**Figure 1:**
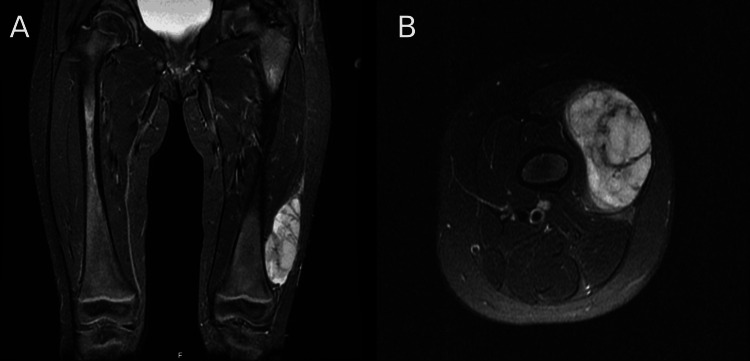
A. Coronal view of MRI shows a well-defined hyper-intense soft tissue mass with marked enhancement at the lateral lower left thigh. B. Axial view of MRI shows no evidence of local bone invasion

A three-year-old girl presented to the orthopedic department with progressive mass swelling in her left thigh for four months as noted by her mother. She had no significant medical or family history. Physical examination revealed a mobile, non-tender, firm mass involving the left lateral distal thigh. MRI imaging showed a 7 cm markedly enhancing, well-defined solid soft tissue mass that abutted the lateral aspect of the lower vastus lateralis muscle (Figure 1A). There was no evidence of local bone invasion or distant metastasis (Figure 1B). She underwent ultrasound-guided biopsy and histopathology showed a spindle cell sarcoma of at least intermediate grade. Later, a wide local resection of the mass was performed and sent for histopathological assessment. 

**Figure 2:**
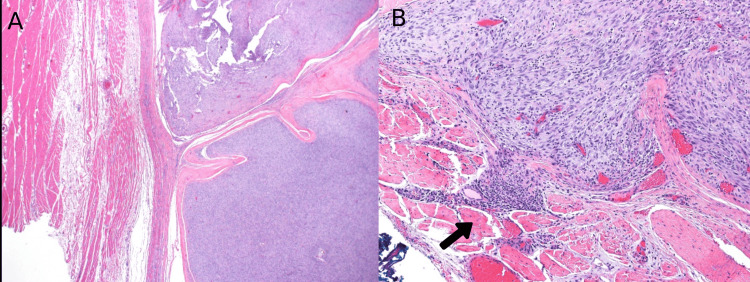
A. Cellular well-circumscribed neoplasm (H&E x4). B. Focal infiltration into adjacent fibroconnective tissue (arrow) (H&E x10).

**Figure 3:**
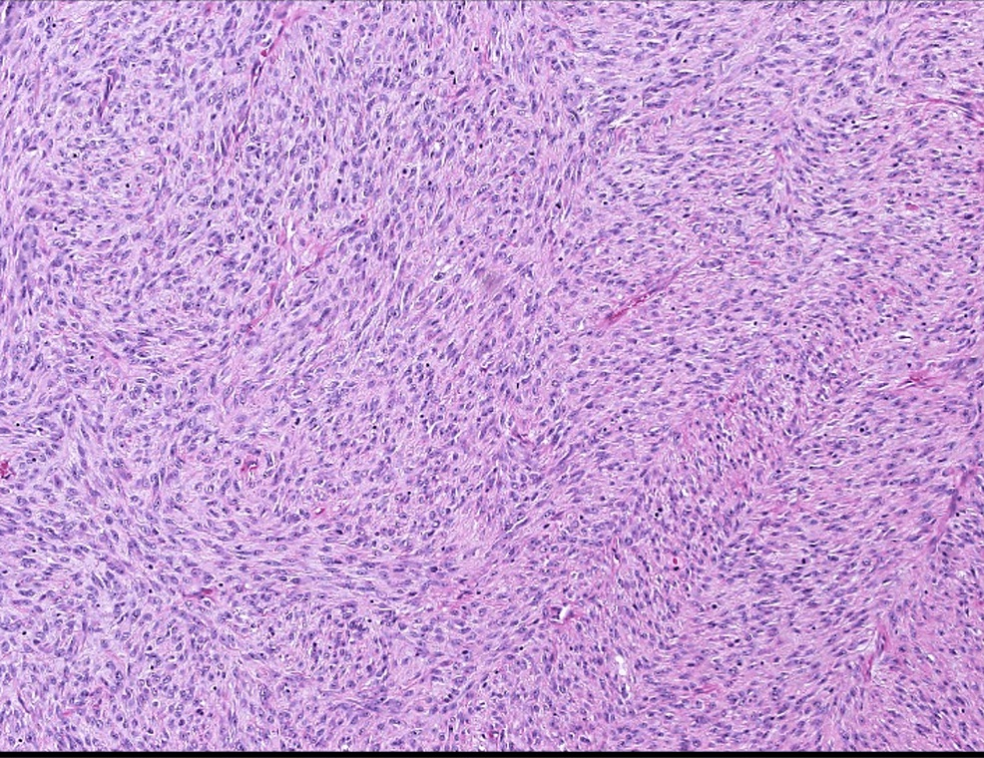
Intermediate power view. Areas of high cellularity with spindle cells arranged in short intersecting fascicles (H&E x10).

**Figure 4:**
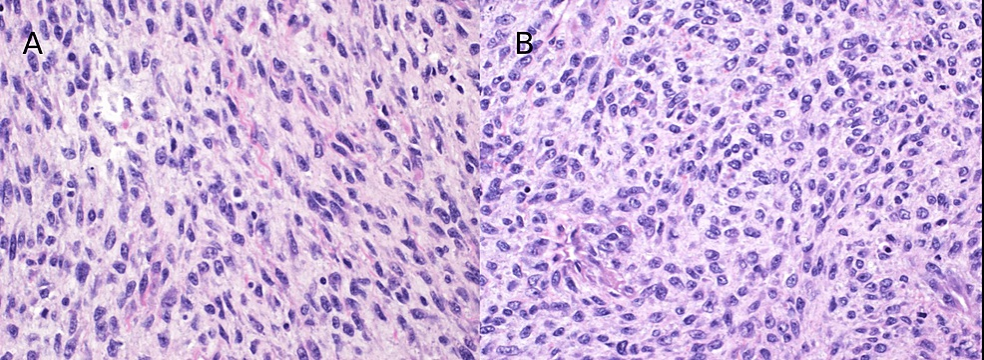
High power view: The tumor is composed of spindle (A) to more ovoid (B) cells with uniform nuclei and inconspicuous nucleoli in a collagenized stroma (H&E x40).

**Figure 5:**
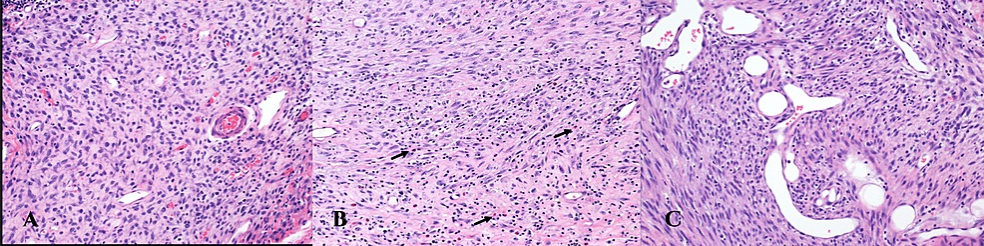
A. Small thin-walled vascular channels with chronic inflammatory infiltrate composed of lymphocytes. (B) Occasional eosinophils (arrow). (C) Markedly dilated and irregular blood vessels (H&E x40).

**Figure 6:**
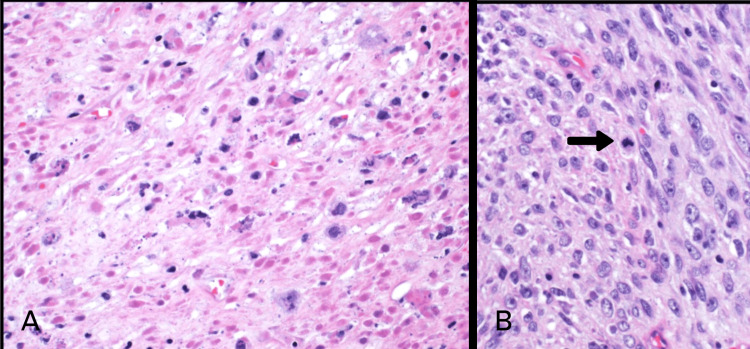
A. Foci of necrosis. B. Mitotic figures easily identified (arrow) (H&E x40).

On gross examination, the mass appeared well-circumscribed and lobulated with areas of myxoid degeneration. It measured 7.5 x 5 x 4 cm. Scanning power microscopy demonstrated a non-encapsulated densely cellular neoplasm with focal infiltration into adjacent fibroconnective tissue (Figure 2A, 2B). The tumor was composed of monomorphic cells, which were spindle to ovoid in shape and arranged in short intersecting fascicles (Figure 3). On higher magnification, the tumor cells were seen to have uniform nuclei with variable nucleoli embedded in the collagenous stroma (Figure 4A, 4B). In some areas, the tumor cells were arranged haphazardly and contained ectatic/angulated thin-walled blood vessels (Figure 5A, 5C). Focal tumor-infiltrating lymphocytes were noted with occasional eosinophils (Figure 5A, 5B, 5C). Mitotic figures were readily seen (11 per 10 high-power fields) with scattered foci of necrosis (Figure 6A, 6B). By immunohistochemical studies, the tumor cells were focally positive for SMA and negative for S100 protein, CD34, Desmin, EMA, AE1/AE3, Myogenin, and Myod-D1. The fluorescent in situ hybridization (FISH) was negative for the gene rearrangement of *ETV6* and *SYT*. NGS was performed on formalin-fixed, paraffin-embedded tissue and revealed a *KIAA1549-BRAF* gene fusion.

No further treatment or chemotherapy was given after the surgery. Follow-up data showed no evidence of local recurrence or distant metastasis for up to three years after complete surgical resection of the mass.

## Discussion

Infantile fibrosarcoma is a locally aggressive, infrequently metastatic tumor that accounts for approximately 5%-10% of all diagnosed sarcomas [11]. Novel pediatric fusion-positive sarcomas have been described in recent literature, including *BRAF* [8]. Due to the rarity and heterogeneity of pediatric sarcomas, an accurate diagnosis and effective treatments may be provided by the identification of genomic profile. Before RNA sequencing by NGS, the *KIAA1549-BRAF* fusion-positive tumor was challenging to diagnose. Here, we present a case of pediatric spindle cell sarcoma with *KIAA1549-BRAF* gene fusions and morphologically overlapping with IFS.

The histopathologic criteria to diagnose IFS usually include infiltrative growth of monomorphic spindle cells composed of mitotically active, fibroblastic spindle cells arranged in cellular sheets and fascicles, with variable lymphocytic infiltrate and hemangiopericytoma-like vascular pattern [3]. Our patient with *KIAA1549-BRAF* spindle cell sarcoma had heterogenous features found in IFS which made the diagnosis more difficult. Another histologic finding in *KIAA1549-BRAF* rearranged sarcoma, in contrast to IFS, was evidence of relatively well-circumscribed borders. Therefore, in a child with *KIAA1549-BRAF* rearranged sarcoma, neither the absence nor the presence of some histologic features found in IFS can be used as a reliable histologic criteria to differentiate these two entities.

Clinically, unlike IFS which commonly involves the extremities, *BRAF-*rearranged sarcomas commonly arise in axial location and have been reported in older children, adolescents, and adults [3,5,7,12]. Our patient with *KIAA1549-BRAF* spindle cell sarcoma presented with thigh mass which was found in two patients according to previous reports [9,10]. Indeed, only one patient with *KIAA1549-BRAF* translocation sarcoma in the previous study had evidence of neurofibromatosis type 2, at the time of presentation [10]. In contrast, an adult patient with the same gene fusion has shown a distinct clinical and morphological feature characterized by haphazard spindle cell proliferation with thin-walled vascular channels [7].

Immunohistochemically, the previous cases of spindle cell sarcomas with *KIAA1549-BRAF* alteration have reported focal expression of Desmin while other markers such as Pan-cytokeratin (AE1/AE3), SMA, Myogenin, CD34, and S100 were all negative [9,10]. Patchy expression of SMA was observed in our case, while other immunostains were negative. The case arising in an adult patient was positive for S100 and CD34 [7]. BRAF V600E immunohistochemistry is sensitive and specific for BRAF V600E mutations in carcinoma and melanoma. However, it is expected to be negative in BRAF-fusion-positive carcinomas and sarcomas. Therefore, immunohistochemical staining for BRAF V600E is not good as a screening marker for fusions involving BRAF [5].

Given the spindle cells appearance of our case and lower limb location, the differential diagnosis includes not only infantile fibrosarcoma but also monophasic synovial sarcoma, spindle cell/ sclerosing rhabdomyosarcoma, malignant peripheral nerve sheath tumor and the emerging entity *NTRK-*rearranged neoplasm with co-expression of S100 and CD34. The combination of our immunohistochemical, genetic, and molecular findings was consistent with a *KIAA1549-BRAF-*rearranged spindle cell sarcoma diagnosis.

BRAF encodes proteins belonging to the RAF family of serine/threonine protein kinases, which have a role in regulating the MAP kinase/ERK signaling pathway and promoting cell survival, proliferation, and transformation [13]. Activating *BRAF* mutations by fusion and point mutations have been reported in various tumor types, such as thyroid carcinoma and melanoma [14]. A significant number of *BRAF*-associated gene fusions have emerged more recently as a result of intrachromosomal gene fusion. However, mesenchymal tumors rarely have *BRAF* gene rearrangements and were recently reported in a subset of spindle cell sarcomas [8]. *KIAA1549* is a novel fusion partner for *BRAF* in soft tissue tumors. *KIAA1549 *is a protein-coding gene belonging to the UPF0606 family. *KIAA1549-BRAF* translocation commonly occurs in a low-grade pediatric central nervous system tumor [15].

We as well as Fujikawa et al. and Nagy et al. have observed that pediatric *KIAA1549-BRAF* rearranged sarcoma is usually a localized tumor with indolent biological behavior and generally treated with initial tumor resection and/or chemotherapy [9,10]. There have been no published reports of this tumor metastasizing to other organs or spreading to the lymph nodes, which points to a favorable outcome. However, adult patients with KIAA1549-BRAF fusion tumors show aggressive clinical behavior, such as distant metastases [7].

With the expanding availability of kinase inhibitor therapies, the presence of a *BRAF* fusion gene has potential therapeutic significance [5]. *MEK* inhibitors and/or dual *MEK/BRAF* inhibition are currently used as targeted therapies for tumors with *KIAA1549-BRAF* fusion and have shown promise in clinical trials [16,17,18]. The use of chemotherapy alone as a therapeutic approach in soft tissue tumors is well-established. In an adult patient, a high-grade spindle cell sarcoma with *KIAA1549-BRAF* that was resistant to conventional chemotherapies was effectively treated with sorafenib, temsirolimus, and bevacizumab as a combination therapy [7]. In our case, surgical resection showed a good prognosis with no local recurrence. However, more studies are needed to establish the proper management for such cases, including the efficacy of *BRAF*-targeted therapy, particularly *MEK* inhibitors for *BRAF*-altered spindle cell sarcomas that morphologically resemble IFS. 

## Conclusions

*KIAA1549-BRAF-*rearranged spindle cell sarcoma is a malignant, yet indolent tumor with an IFS-like pattern that is usually composed of spindle cells. The immunohistochemical profile of pediatric *KIAA1549-BRAF-*rearranged spindle cell sarcoma is rather nonspecific, with only a focal expression of SMA or Desmin. For *ETV6-NTRK3-*negative spindle cell sarcomas that morphologically resemble IFS, the confirmation of genomic profile by NGS may help us make an accurate diagnosis and select novel therapeutic options. Future studies are needed to determine the effectiveness of *BRAF*-targeted therapy in theses sarcomas.
